# Apatinib exhibits anti-leukemia activity in preclinical models of acute lymphoblastic leukemia

**DOI:** 10.1186/s12967-018-1421-y

**Published:** 2018-02-28

**Authors:** Manman Deng, Jie Zha, Zhiwu Jiang, Xian Jia, Yuanfei Shi, Peng Li, Xiao Lei Chen, Zhihong Fang, Zhiqiang Du, Bing Xu

**Affiliations:** 1 Department of Hematology, The First Affiliated Hospital of Xiamen University and Institute of Hematology, Medical College of Xiamen University, Xiamen, 361003 People’s Republic of China; 2 Key Laboratory of Regenerative Biology, Southern China Institute for Stem Cell Biology and Regenerative Medicine, Guangzhou Institutes of Biomedicine and Health, Chinese Academy of Sciences, Guangzhou, China; 3 State Key Laboratory of Cellular Stress Biology, Innovation Center for Cell Signalling Network, School of Life Sciences, Xiamen University, Xiamen, 361005 China; 4 Department of Translational Science, Amgen Asia R&D Center, Shanghai, China

**Keywords:** Apatinib, Acute lymphoblastic leukemia, VEGFR2, Leukemia therapy, Anti-angiogenic agent

## Abstract

**Background:**

Acute lymphoblastic leukemia (ALL) is a clonal malignant disorder characterized by an uncontrolled proliferation of immature B or T lymphocytes. Extensive studies have suggested an involvement of angiogenesis signaling in ALL progression and resistance to treatment. Thus, targeting angiogenesis with anti-angiogenic drugs may be a promising approach for ALL treatment. In this study, we investigated the effectiveness of Apatinib, a novel receptor tyrosine kinase inhibitor selectively targeting VEGFR-2 in ALL cells.

**Method:**

ALL cell lines were treated with different concentration of Apatinib and then CCK8 assay, flow cytometry were used to determine the IC_50_ value and cell apoptosis, respectively. The effect of Apatinib against primary ALL cells from 11 adult patients and normal counterparts were also analyzed by apoptosis with flow cytometry. Next, we used western bolting and mass cytometry (CyTOF) assay to explore the underlying mechanism of the cytotoxicity of Apatinib. Finally, the anti-leukemia activity was further evaluated in an in vivo xenograft model of ALL.

**Results:**

Our results showed that Apatinib significantly inhibited cell growth and promoted apoptosis in both B and T lineage ALL cell lines in a dose- and time-dependent manner. The IC_50_ values of Apatinib against Nalm6, Reh, Jurkat and Molt4 for 48 h were 55.76 ± 13.19, 51.53 ± 10.74, 32.43 ± 5.58, 39.91 ± 9.88 μmol/L, and for 72 h were 30.34 ± 2.65, 31.96 ± 3.92, 17.62 ± 5.90, and 17.65 ± 2.17 μmol/L respectively. Similarly, Apatinib shows cytotoxic activity against primary adult ALL cells while sparing their normal counterparts in vitro. Moreover, Apatinib suppressed ALL growth and progression in an in vivo xenograft model. Mechanistically, Apatinib-induced cytotoxicity was closely associated with inhibition of VEGFR2 and its downstream signaling cascades, including the PI3 K, MAPK and STAT3 pathways.

**Conclusion:**

Our study indicates that Apatinib exerts its anti-leukemia effect by inducing apoptosis through suppressing the VEGFR2 signaling pathway, supporting a potential role for Apatinib in the treatment of ALL.

**Electronic supplementary material:**

The online version of this article (10.1186/s12967-018-1421-y) contains supplementary material, which is available to authorized users.

## Background

Acute lymphoblastic leukemia (ALL) is caused by malignant transformation and proliferation of lymphoid progenitor cells of either B or T cells. In children, ALL patients enjoy an approximately 90% long-term survival with the current pediatric chemotherapy protocols [1]. However, ALL is a devastating disease when it occurs in the adults, with reportedly only 30–40% long-term survival rate [2]. Despite many advances in management, the backbone of ALL therapy remains as multi-agent chemotherapy with vincristine, corticosteroids, cyclophosphamide and anthracycline, and may involve allogeneic stem cell transplantation for eligible candidates [2]. While dose intensification strategies have led to a significant improvement in outcomes for pediatric patients, the elderly patients usually do not benefit from or sometimes can be even over-treated by these therapies, as they are unable to tolerate such regimens and consequently may carry an extremely poor prognosis. Therefore, there is an urgent need to develop additional therapeutic regimens for adult ALL patients.

The essential role of angiogenesis, a process of blood vessel formation, in the growth and maintenance of solid tumors was well established. Over the past decades, emerging evidence also suggests an involvement of angiogenesis in leukemogenesis and leukemia progression [3]. Increased angiogenesis in bone marrow niche correlates with both acute lymphoblastic leukemia and acute myeloid leukemia progression and resistance to treatment [4–8]. Moreover, a recent study identified an important role of the VEGF/VEGFR2 axis in regulating ALL cell migration through the cerebral microvascular endothelial barrier, contributing to central nervous system (CNS) leukemia [9]. Thus, targeting angiogenesis with anti-angiogenic drugs or VEGFR inhibitors may be a new approach for ALL treatment. Apatinib, also known as YN968D1, is an orally administrated small molecule receptor tyrosine kinase inhibitor selectively targeting VEGFR-2 [10]. It was approved in China as a subsequent-line treatment for patients with advanced gastric cancer [11], and has been tested for many other types of cancers in phase II/III clinical trials in China, such as non-small-cell lung cancer, breast cancer, and hepatocellular carcinoma [12]. Promising results obtained from these clinical trials demonstrated that Apatinib has anti-tumor activity across a broad range of advanced cancers. This prompts us to investigate whether Apatinib can be used for ALL treatment and the potential underlying mechanisms.

## Methods

### Reagents

Apatinib was kindly provided by Jiangsu Hengrui Medicine Company (Jiangsu, China) and dissolved in DMSO (Invitrogen, USA) as a 100 mM stock solution. It was diluted to desired concentrations in subsequent experiments with growth media.

### Cell lines and cell culture

The following human B and T lineage acute lymphoblastic leukemia cell lines were used in this study: Nalm6, Reh, Molt4 and Jurkat. Cells were cultured in RPMI-1640 (Hyclone, Thermo Scientific, MA, USA) supplemented with 10% fetal bovine serum (FBS, Gibco, Life Technologies, NY, USA) and 100 units/mL penicillin and 100 μg/mL streptomycin at 37 °C in a water-saturated atmosphere with 5% CO_2_.

### Primary samples

Mobilized peripheral blood mononuclear cells were obtained from healthy donors for hematopoietic stem cell transplantation with a standard 5 day G-CSF treatment. Bone marrow samples with B or T lineage acute lymphoblastic leukemia were obtained from patients after informed consent in accordance with the guidelines of the first affiliated hospital of Xiamen University. The study protocols were approved by the Ethics Committee and accorded with the Declaration of Helsinki. Mononuclear cells were isolated by standard Ficoll-Hypaque density centrifugation (BD, Franklin Lakes, NJ, USA) and cultured in IMDM [HyClone, Thermo Scientific, MA, USA) supplemented with 10% FBS (Gibco, Life Technologies, NY, USA)], 100 U/mL penicillin and 100 µg/mL streptomycin (1 × P/S) at 37 °C.

### In vitro cell viability assay

Cytotoxicity of Apatinib against ALL cell lines was determined using a CCK8 kit (Dojindo, Kumamoto, Japan). Briefly, cells were first aliquot into 96-well plates at 5 × 10^4^ cells/well with 100 μL of growth medium and then treated with designated dosages of Apatinib for 48 or 72 h. After that CCK-8 reagents (10 µL/well) were added and cells were further incubated for 2 h in the incubator. Finally, absorbance at 450 nm was read by a microplate reader (ELX800, Bio TEK, USA). All experiments were repeated at least three times with triplicate in each experiment. The IC_50_ value (half maximal inhibitory concentration) of each cell line was calculated using the GraphPad Prism 5 software.

### Apoptosis assay

Annexin V and Propidium iodide (PI) (Ebioscience, San Diego, USA) dual staining was performed to detect apoptotic cells following manufacturer’s instructions. Briefly, cells (2 × 10^5^ cells/well) were seeded into 24-well plates and treated with different concentrations of Apatinib for 48 or 72 h. Then cells were harvested, washed twice with ice-cold PBS, double-labeled with Annexin V/PI for 30 min at 4 °C in the dark, followed by analysis with flow cytometry using the FACS C6 software (BD, Oxford, UK).

### Western blot analysis

Whole cell lysates (50 μg/line) from each sample were electrophoresed in 8% or 10% SDS-PAGE and transferred to PVDF membrane (Millipore, Billerica, MA, USA). After blocking for 1 h for non-specific binding in TBS-T with 5% non-fat milk, membranes were incubated with primary antibodies [VEGFR, rabbit monoclonal, 1:1000; p-VEGFR, rabbit monoclonal, 1:1000; p-AKT (ser473), rabbit monoclonal, 1:1000; AKT, rabbit monoclonal, 1:1000; p-ERK, rabbit monoclonal, 1:1000; ERK, rabbit monoclonal, 1:1000; GAPDH, rabbit monoclonal, 1:1000; β-actin, rabbit monoclonal, 1:1000, Cell Signaling Technology, Inc. Herts, UK] overnight at 4 °C and followed by secondary HRP-conjugated monoclonal antibody (1:10000, Cell Signaling Technology) for 1 h at room temperature. The expression of target proteins was detected by enhanced chemiluminescence western blotting detection kit (Amersham, Little Chalfont, UK) following the manufacturer’s instructions.

### Mass cytometry (CyTOF) analysis

Bone marrow mononuclear cells from one patient with B-ALL was collected and used for the Mass Cytometry (CyTOF) analysis. Briefly, cells with 95% or higher viability were treated with 0 or 20 μM of Apatinib for 48 h and washed three times with washing buffer (phosphate-buffered saline plus 0.5% bovine serum albumin). Samples of 3 × 10^6^ cells were incubated with 0.75 μM 195 Pt monoisotopic cisplatin (Fluidigm, San Francisco, CA) at room temperature for 2 min, followed by staining with the mass cytometry antibody cocktail against surface and intracellular proteins (Additional file 1: Table S1) following the manufacturer’s protocol. Cells were subsequently washed and stained with 125 nM Iridium intercalator (Fluidigm, San Francisco, CA) overnight at 4 °C. Lastly, cells were washed twice with H_2_O and re-suspended in 500 μL H_2_O for CyTOF analysis. Helios™ Software V6.5.358 acquisition software (Fluidigm, San Francisco, CA) was used to normalize to internal bead standards. Data were evaluated using Cytobank software (Cytobank Inc.).

### Human acute lymphoblastic leukemia xenograft experiments

NODscid-IL2Rg−/− (NSI) mice, which has a high tumor engraftment index (TEI) score [13], were used to establish ALL xenografted model according to our previous study [14]. For briefly, 6–8 week old male were provided by Guangzhou Institutes of Biomedicine and Health (GIBH) and housed under specific-pathogen-free (SPF) conditions following the animal care guidelines. The protocols for the animal studies were approved by the Animal Welfare Committee of GIBH. Before inoculation, mice received 1 Gy of total body irradiation at a dose rate of 325 cGy/min by parallel opposed 4 MV X-ray. Within 24 h, mice were intravenously injected via retro-orbital vein with 5 × 10^6^ ALL cells (Nalm6 cell line). Seven days post transplantation of ALL cells, mice were randomly assigned to either control or Apatinib treatment group (n = 6 per group), and treated with either vehicle (PBS and 0.5% methyl cellulose/0.5% Tween 80 in PBS) or 100 mg/kg/day Apatinib by oral gavage from Monday to Friday for 2 consecutive weeks. At the end of the experiments, mice were euthanized and their spleens were photographed and weighed. The leukemia load (human CD45+ cells) in peripheral blood (PB), bone marrow (BM) and spleen was determined by flow cytometry staining with anti-human-CD45 antibody. The bone marrow and spleen were fixed in 10% paraformaldehyde for 24 h, followed by paraffin-embedding and H&E staining.

### Statistical analysis

Data were represented as mean ± S.D of at least three independent experiments. The statistical analyses were performed using the SPSS 19.0 and GraphPad Prism 5.0 software. Comparison between two groups were analyzed by 2-tailed Student’s *t* test. Statistical analyses of multiple-group comparisons were performed by one-way analysis of variance (ANOVA) followed by the Bonferroni posthoc test. *P* values < 0.05 was considered as statistically significant.

## Results

### Apatinib inhibits B and T lineage ALL cell growth in a dose- and time-dependent manner

**Fig. 1:**
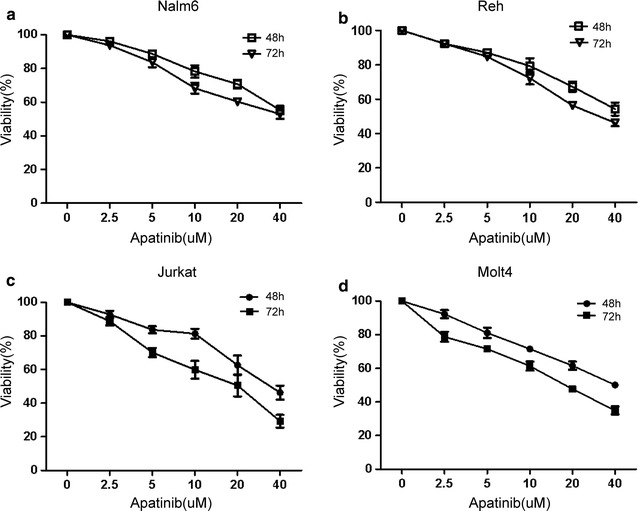
Apatinib exhibits a dose- and time-dependent inhibition of proliferation of B and T-lineage ALL cell lines. B-lineage (**a**, **b**) and T-lineage ALL cells (**c**, **d**) were exposed to indicated concentrations of Apatinib for 48 or 72 h, and cell viability was subsequently determined by a CCK-8 kit

As an approved drug for gastric cancer, we wondered whether Apatinib could be effective in leukemia equally. We tested the anti-leukemic effect of Apatinib in four ALL cell lines: Nalm6, Reh, Jurkat and Molt4. We used the Cell Counting Kit-8 (CCK8) assay to examine the cytotoxic effect of Apatinib on two human B-ALL cell lines (i.e., Nalm6 and Reh) and two human T-ALL cell lines (i.e., Jurkat and Molt4). In this assay, water-soluble tetrazolium salt WST-8 is reduced by dehydrogenases within living cells and subsequently being converted into orange colored formazan, of which the amount of the dye directly proportional to the number of living cells. As shown in Fig. 1, Apatinib remarkably inhibited cell proliferation of all these four cell lines in a dose- and time-dependent manner. After treatment for 48 h, the IC_50_ values of Nalm6, Reh, Jurkat and Molt4 were 55.76 ± 13.19, 51.53 ± 10.74, 32.43 ± 5.58 and 39.91 ± 9.88 μM, respectively. As expected, the IC_50_ values of 72 h were lower than that of 48 h (Table 1). Moreover, the IC_50_ for Apatinib against T-ALL cells was lower than that for B-ALL, suggesting that Apatinib was more effective in inhibiting the growth of T-lineage leukemia cells.

**Table 1:** The IC_50_ values of Apatinib in treating B- and T-lineage ALL cell lines

All cell lines	IC_50_ ± S.D (μmol/L)		*P* value
	48 h	72 h	
Nalm6	55.76 ± 13.19	30.34 ± 2.65	0.031
Reh	51.53 ± 10.74	31.96 ± 3.92	0.041
Jurkat	32.43 ± 5.58	17.62 ± 5.90	0.034
Molt4	39.91 ± 9.88	17.65 ± 2.17	0.019

### Apatinib induces apoptosis of both B and T lineage ALL cells

**Fig. 2:**
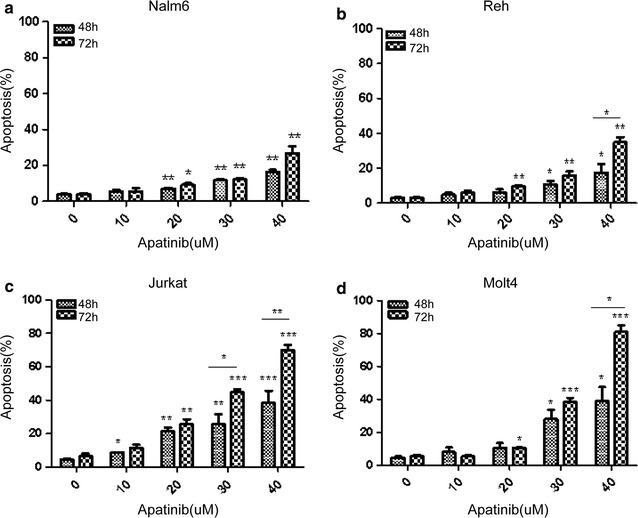
Apatinib induces apoptosis of both B and T lineage ALL cells in a dose and time-dependent manner. B-lineage (**a**, **b**) and T-lineage ALL cells (**c**, **d**) were treated with indicated doses of Apatinib for 48 or 72 h, and the percentage of apoptotic cells was examined by Annexin V/PI double staining. *P < 0.05; **P < 0.01; ***P < 0.001

Apatinib is a small molecule inhibitor of receptor tyrosine kinase, we suspected that Apatinib may inhibit the proliferation of ALL cell lines by triggering cell death. Thus we examined the cell death status of B or T-ALL cell lines after Apatinib treatment. Nalm6, Reh, Jurkat and Molt4 cells were treated with indicated concentrations of Apatinib for 48 or 72 h, stained with Annexin V and PI. and finally subjected to flow cytometric analysis. Apatinib treatment resulted in significantly enhanced apoptosis in each cell line tested in a dose-dependent manner when compared to their untreated controls (Fig. 2a–d). Similar to the observation on cell growth inhibition (Fig. 1), Apatinib-induced apoptosis was more pronounced in T-ALL cells than in B-ALL cells (Fig. 2). Taken together, these data suggests that induction of apoptosis might contribute to the encouraging anti-leukemia effect of Apatinib.

### Apatinib shows preferential cytotoxic activity against primary adult ALL cells but not normal PBMCs

**Fig. 3:**
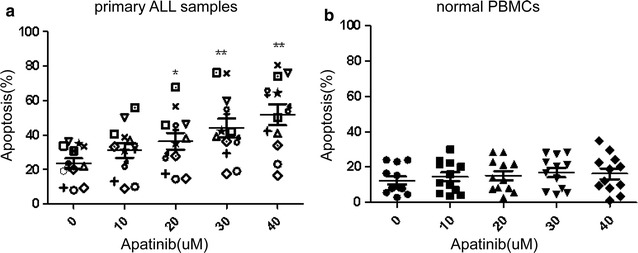
Apatinib shows preferential cytotoxic activity against primary adult ALL cells while sparing normal PBMCs. The percentage of apoptotic cells (Annexin V+) of primary ALL patients (**a**) and peripheral blood or bone marrow of healthy donors (**b**) after exposure to indicated doses of Apatinib for 48 h. *P < 0.05; **P < 0.01

To further confirm the anti-leukemia activity of Apatinib, primary samples (bone marrow mononuclear cells) from 11 adult patients with B- or T-ALL were treated with Apatinib. Clinical characteristics of these B- or T-ALL patients are summarized in Table 2. Consistent with the anti-leukemia effect observed in ALL cell lines, exposure of primary ALL cells to Apatinib resulted in remarkable apoptosis in a dose dependent manner (Fig. 3a). In contrast, Apatinib displayed minimal, if any, toxicity towards normal peripheral blood mononuclear cells (PBMCs) collected from healthy donors of hematopoietic stem cell transplantation (HSCT) under the same conditions (Fig. 3b). These findings highlighted a specific cytotoxic activity of Apatinib towards ALL cells.

**Table 2:** Clinical characteristics of ALL patients (n = 11)

Patient No.	Age (years)	Gender	T or B-ALL	Cell count (×10^9^/L)			LDH (U/L)	Extramedullary infiltration	Karyo-type	Molecular features
				WBC	HB (g/L)	PLT				
1	31	F	B	12.29	75	32	349	Yes	Complex	MYC,P16,E2A
2	18	M	B	33.64	81	65	330	Yes	46,XY	IGH5′
3	29	F	T	286.68	58	71	362	Yes	NA	–
4	22	M	T	3.87	137	305	162	Yes	NA	–
5	21	M	B	2.85	63	9	542	No	46,XY	IGH
6	71	M	B	141.25	102	85	590	Yes	NA	NA
7	58	F	B	124	82	19	4252	Yes	t(4;11)(q25;q22)	MFF/AF4
8	36	M	B	51.62	96	59	283	Yes	Complex	–
9	23	M	T	247.36	66	37	4623	Yes	NA	P16,E2A,IGH
10	59	M	B	62.97	101	11	395	Yes	t(9;22)(q34;q11)	BCR/ABL,P16
11	53	M	B	87.02	115	9	1521	Yes	t(9;22)(q34;q11)	BCR/ABL

### Apatinib down-regulates the phosphorylation of VEGFR2 and its downstream signaling pathways in ALL cells

**Fig. 4:**
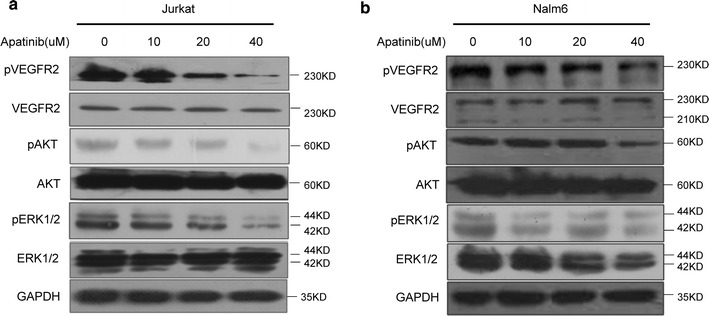
Apatinib down-regulates the phosphorylation of VEGFR2 and its downstream signaling pathway in ALL cells. Jurkat (**a**), Nalm6 (**b**) cells were treated with 0, 10, 20 and 40 μM Apatinib for 48 h, respectively. The protein level of VEGFR2, p-VEGFR2 and its downstream signaling pathways were examined by western blotting. β-actin and GAPDH was used as a loading control in this experiment

**Fig. 5:**
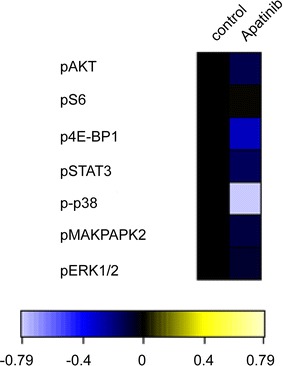
The effect of Apatinib on intracellular signaling pathways was examined by CyTOF at the single-cell level in primary ALL. The primary ALL cells were exposed to 20 μM Apatinib for 48 h, and subjected to CyTOF analysis of intracellular signaling pathways downstream of VEGFR2

To further investigate the mechanism of action how Apatinib kill ALL cells, phosphorylated-VEGFR2 (p-VEGFR2) was analyzed by western blot in ALL cell lines. As shown in Fig. 4, Apatinib markedly diminished the phosphorylation of VEGFR2 in Nalm6 and Jurkat cells in a dose-dependent manner, consistent with its ability to induce cell death. Moreover, VEGFR2 downstream signals, p-ERK and p-AKT, were also down-regulated (Fig. 4a, b). To further pinpoint the mechanism underlying Apatinib killing of ALL leukemia cells, we used mass cytometry (CyTOF) to measure intracellular signaling pathways at the single-cell level. Our results showed that multiple signaling pathways downstream of VEGFR2 were repressed by Apatinib, including the PI3K (AKT, S6, 4E-BP1), MAPK (p38, MAPKAPK2, ERK1/2) and STAT3 pathways (Fig. 5). Together, these findings suggest that Apatinib induced ALL cell death by inhibiting the phosphorylation of VEGFR2 and its downstream signaling pathways.

### Apatinib suppresses leukemia cell growth and leukemia progression in an in vivo xenograft model

**Fig. 6:**
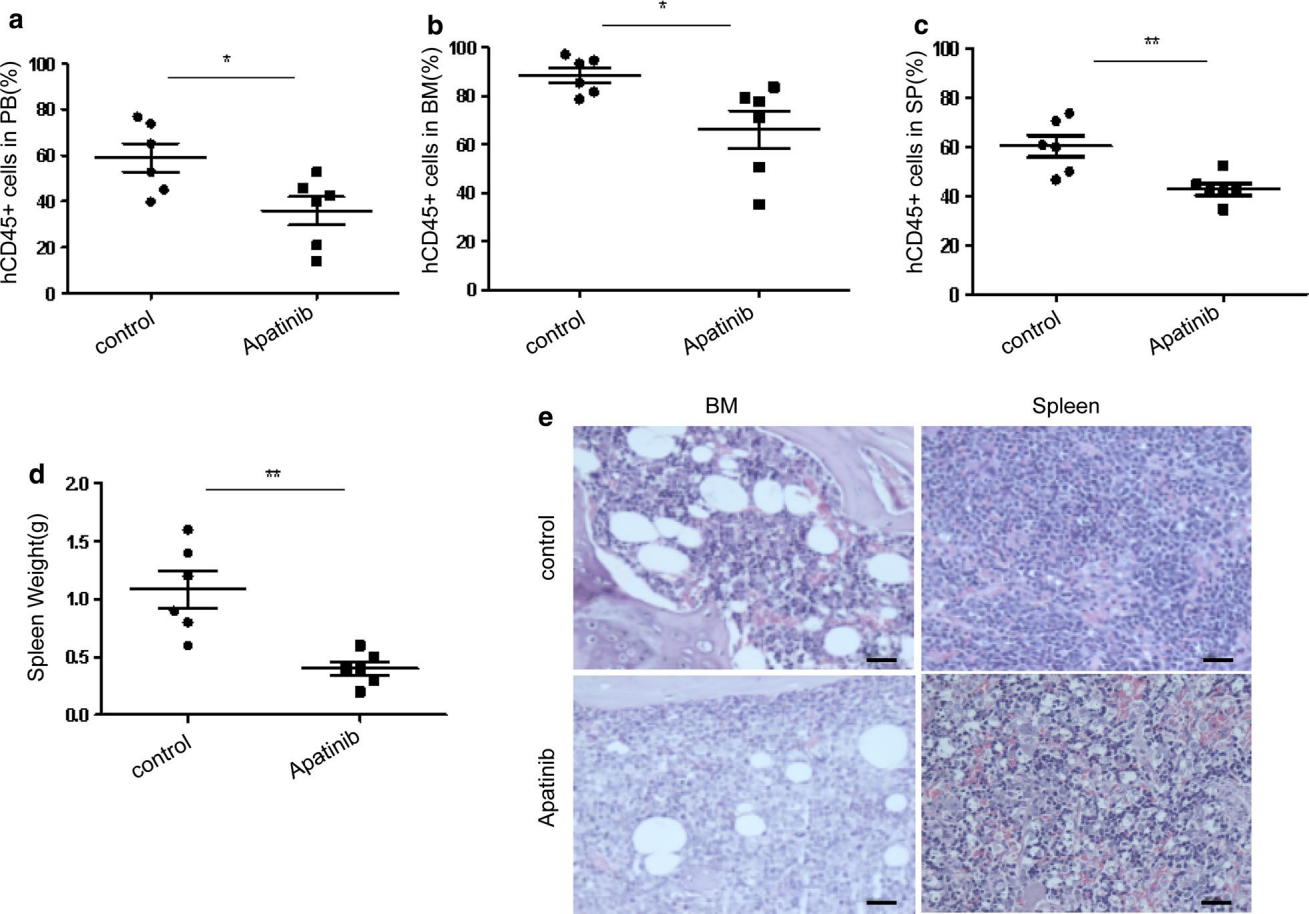
Apatinib suppresses leukemia cell growth and leukemia progression in a xenograft model. Nalm6 cells were intravenously injected into NSI mice. 7 days later, mice were randomized (n = 6 per group) and treated with vehicle (control group) or Apatinib (administered by oral gavage at the dose of 100 mg/kg for 2 consecutive weeks). Percentage of human CD45+ (hCD45) cells in peripheral blood (**a**), bone marrow (**b**) and spleen (**c**) were determined by flow cytometry. **d** Spleens of mice were weighted at the end of the experiment. **e** Spleen and bone marrow of mice were embedded with paraffin and stained with H&E. Scale bar, 25 μm.*P < 0.05; **P < 0.01

To further evaluate the anti-leukemia activity of Apatinib in vivo, we established an acute lymphoblastic leukemia xenograft models by intravenous injection of Nalm6 cells into NODscid-IL2Rg−/− (NSI) mice. Prior to drug treatment, the leukemia burden of mice was measured by analyzing the percentage of hCD45 positive cells in peripheral blood, documenting no significant difference in baseline tumor burden between control and treatment groups (Additional file 2: Figure S1). Apatinib was administered by oral gavage once a day with a dose of 100 mg/kg/day, from Monday to Friday for consecutive 2 weeks. Mice treated with Apatinib showed a remarkable reduction in leukemia burden, manifested by a significant decrease in the numbers of human CD45^+^ cells in the peripheral blood, spleen and bone marrow as compared to control mice (Fig. 6a–c). Moreover, the average weight of spleens in mice treated with Apatinib was significantly lower than that of control mice (Fig. 6d). Furthermore, histopathology analysis indicated that Apatinib significantly prevented the infiltration of leukemic cells into the bone marrow and spleen (Fig. 6e).

## Discussion

Angiogenesis plays a critical role in the growth, progression, and metastasis of cancers [15], including hematological leukemia [3]. Vascular endothelial growth factor (VEGF) and its receptors (VEGFRs) function as key regulators of this process. The VEGFR-family proteins consist of three members: VEGFR-1 (Flt-1), VEGFR-2 (KDR/Flk-1), and VEGFR-3 (Flt-4), among which VEGFR-2 is thought to be principally responsible for angiogenesis in malignancies [16]. Inhibition of VEGF/VEGFRs mediated signaling shows promise as an anti-angiogenic strategy for solid tumors [17]. Several small molecule VEGF/VEGFR pathway inhibitors have been approved for the treatment of metastatic colorectal cancer, gastrointestinal stromal tumors, hepatocellular carcinoma, non-small-cell lung cancer, renal cell carcinoma, soft tissue sarcoma, and medullary thyroid cancer, and are under the development of a number of other oncology indications, including pancreatic cancer, ovarian cancer, breast cancer, prostate, head and neck cancers [17]. These anti-angiogenic drugs have improved progression-free survival and in some cases overall survival for several cancer types. However, clinical experience with the current anti-angiogenic agent, Apatinib, remains limited in treating aggressive leukemias, especially ALL [18]. Therefore, the development of new drugs targeting angiogenesis may open up a new era of anti-angiogenic combination therapy of human leukemia. In the present study, we showed that a novel anti-angiogenic drug, Apatinib, inhibited both B and T lineage ALL cell growth and progression in in vitro and in vivo preclinical models.

Apatinib is a potent tyrosine kinases inhibitor of VEGFR-2, PDGFR-β, RET, c-Src and c-Kit [10, 19, 20]. It can effectively inhibit the proliferation, migration and tube formation of human umbilical vein endothelial cells (HUVEC), block the microvessel budding of rat aortic ring, and show anti-tumor efficacy in vivo against a variety of established tumor xenografts with good tolerance [8]. Moreover, Apatinib could reverse ABCB1- and ABCG2-mediated multidrug resistance in solid tumors as well as in leukemia cell lines [21, 22], thereby enhancing the efficacy of chemotherapeutic agents. Based on this mechanism, Apatinib has proved to be effective and safe in the treatment of advanced gastric cancer patients [11, 12]. However, anti-tumor activity of Apatinib in ALL had not been explored. Here, we showed that Apatinib not only suppresses the proliferation but also induces apoptosis of ALL cells, with low cytotoxicity towards normal hematopoietic cells. Our results may extend the field of indications for Apatinib to the treatment of leukemia. As expected, we found that primary leukemia cells from adult ALL patients are all sensitive to Apatinib in cell culture. Currently, 750 mg once daily of Apatinib mesylate is recommended for clinical studies in patients with advanced solid tumors and well tolerated [23]. Pharmacokinetic analysis showed a peak plasma concentration (C_max_) of 3.8–4.0 μM was achieved in patients continuously treated with this dose of Apatinib [24]. In the present study, the IC_50_ of Apatinib varied in a range of 17–56 μM in vitro in this setting directly targeting ALL cells, while it could be active at concentration as low as 2.5 μM, which appears to be within the range of C_max_ in vivo. In this context, it has also been demonstrated earlier that Apatinib induces apoptosis and cell cycle arrest of osteosarcoma cells at a concentrations range of 5–20 μM [25]. Moreover, the dose-escalation experiment of Apatinib displayed no obvious toxicity towards normal hematopoietic stem cells, suggesting that this dose range might be tolerated in vivo. Nevertheless, these results raise a possibility that it might require a dose higher than 750 mg daily of Apatinib to be effective in treatment of ALL.

Interestingly, it seems that acute lymphoblastic T leukemias are more susceptible to Apatinib-induced apoptosis in our experiments. A low activity of p-VEGFR in T lymphoblastic leukemia cell lines might account for a relatively lower IC_50_ of Apatinib against T lymphoblastic leukemia cells. In addition, previous studies showed that a high functional P-glycoprotein activity is frequently present in T-ALL [26]. Therefore, Apatinib might block the efflux function of P-glycoprotein in T-ALL cells, rendering T-ALL cells more vulnerable to Apatinib-induced apoptosis.

Extramedullary infiltration is a common phenomenon in relapse ALL even after allogeneic hematopoietic stem cell transplantation, contributing to poor prognosis and mortality [27]. In general, the sites most frequently affected by extramedullary relapse are the testicles and the central nervous system [28, 29]. Relapse in other tissues has also been reported, including kidney, breast and the gastrointestinal system [30–32]. Several molecular pathways are involved in solid tumor metastasis, including the VEGF signaling pathway [33]. However, the mechanism underlying extramedullary infiltration of ALL cells remains poorly understood. More recently, Münch et al. [9] found that VEGF signaling regulates trans-endothelial leukemia cell migration and contributes to extramedullary infiltration of ALL cells. In line with it, the inhibition of VEGF signaling by Apatinib led to a specific effect in vivo, namely significantly decreased leukemia burden in bone marrow and spleen of leukemic mice. On the other hand, high VEGF levels have also been associated with poor prognosis and ALL patients with high VEGF levels after induction therapy relapse earlier than those with low VEGF levels [34]. Accordingly, Apatinib, in combination with conventional therapy, offers the promise of effective anti-leukemia activity, as well as improvements in remission and survival of ALL patients.

The primary mechanism for the anti-tumor effect of Apatinib is the suppression VEGF/VEGFR2 signaling, which activates the PI3K-AKT-mTOR and PLC-ERK1/2 pathways [35, 36]. Similarly, our study indicated that AKT and ERK1/2 inactivation contributes to Apatinib-induced inhibition of cell proliferation and promotion of apoptosis of ALL cells. A recent study suggested that Apatinib promotes autophagy and induces apoptosis through VEGFR2/STAT3/BCL-2 signaling in osteosarcoma [25]. These studies suggest multiple signaling cascades downstream of the VEGF/VEGFR2 signaling. To pinpoint the mechanism underlying Apatinib inhibition of ALL leukemia cells, mass cytometry (CyTOF), a powerful technology able to detect up to 50 different parameters at the single-cell level [37], was used in our study. Our CyTOF analysis of an ALL patient’s primary cells showed that multiple signaling cascades downstream of VEGFR2 were repressed by Apatinib, including the PI3K, MAPK and STAT3 pathways. Collectively, our study demonstrates that Apatinib exerts an anti-leukemia effect by inhibiting VEGFR2 signaling and its multiple downstream signaling cascades.

## Conclusion

Taken together, this study reveals for the first time that Apatinib exerts anti-leukemia activity in several in vitro and in vivo preclinical models of acute lymphoblastic leukemia, likely through inhibiting VEGFR2-mediated signaling pathways, and suggests potential benefits and clinical application of Apatinib in the treatment of ALL patients.

## Additional files

**Additional file 1: Table S1.** The CyTOF antibody panel.

**Additional file 2: Figure S1.** The leukemia burden of xenograft model before treatment.

## Abbreviations

ALL: acute lymphoblastic leukemia
CCK8: Cell Counting Kit-8
MAPK: mitogen activated protein kinase
