# Supplementary figures and images for: Apatinib exhibits anti-leukemia activity in preclinical models of acute lymphoblastic leukemia
Source: J Transl Med. 2018 Feb 28;16:47. doi: 10.1186/s12967-018-1421-y (PMC5831852; doi:10.1186/s12967-018-1421-y)

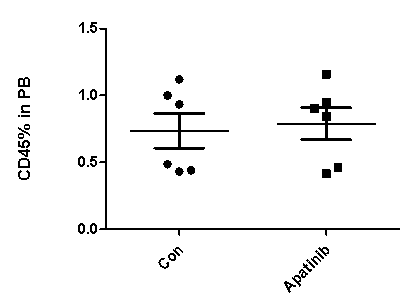

Supplement: Supplementary file 2 — Additional file 2: Figure S1. The leukemia burden of xenograft model before treatment. [file 12967_2018_1421_MOESM2_ESM.tif]
